# A preliminary spatial analysis of diagnosed stroke disease in Osun state, Nigeria

**DOI:** 10.11604/pamj.2016.25.63.5931

**Published:** 2016-10-03

**Authors:** Ayila Emmanuel Adzandeh, John Awope, Osaretin Isoken Oviasu

**Affiliations:** 1 Regional Centre for Training in Aerospace Surveys (RECTAS), Off Road 1, Obafemi Awolowo University campus, Ile-Ife, Osun State, Nigeria

**Keywords:** Spatial analysis, stroke, incidence rate, prevalence

## Abstract

**Introduction:**

There have been a number of clinical studies on diagnosed Stroke disease. However, there have been few studies on the geographical disparities for stroke. This study investigates the spatial pattern of stroke disease reflecting socio-demographic characteristics in the State.

**Methods:**

Stroke patients' admissions for 22 years (from 1990 to 2012) were examined. Their socio-demographic characteristics were extracted from their health records and analyzed. The location of the stroke patients were categorized by Local Governments Areas (LGAs). Spatial maps were generated and produced in a Geographical Information System (GIS) environment. It involves the analysis of the distribution of stroke cases in relation to their underlying population to determine the areas of high and low density of diagnosed cases across the state.

**Results:**

The result highlighted the spatial distribution of diagnosed stroke cases and also highlighted the areas of concern regarding their spatial distribution within the state. Social inequalities in stroke were persistent as incidence rates in urban areas (North) were around 3 times higher than in the rural areas (South). However, this could be due to better healthcare access in the urban areas than in the rural areas as there were disparities in the distribution of healthcare facilities involved in administering care to stroke patients in Osun State.

**Conclusion:**

The outcome of this study appears to indicate that spatial inequalities in the access to Stroke healthcare is a concern that needs to be addressed in order to manage the disease adequately.

## Introduction

The rate at which people suffer from stroke disease in Nigeria is growing. Stroke ranked the number three (3) causes of death in Nigeria even in children, a serious economic burden [1]. Incidence of “Stroke” (Cerebrovascular Accident) in sub-Saharan Africa is alarming, an area where stroke units are largely not feasible and many patients do not reach the hospital [2, 3]. It is also the leading cause of morbidity, mortality and disability in adults of productive ages that contribute the work force of the society [4]. High rates of hypertension, diabetes, alcohol abuse, smoking, insufficient fruit, and vegetables consumption, sickle cell disease, HIV infection, antiretroviral use and race are likely contributing factors [1, 5]. There is evidence of geographical disparities for stroke disease worldwide. For instance, Southeast is reported in the United States with the highest risks [6]. The cluster of determinants of stroke at neighborhood level can greatly affect the planning, implementation and health focus initiatives that attempts to minimize disparities. A Statewide Stroke Incidence Information Registry could offer land administration officials the appropriate spatial information to focus efforts during medical and health care emergencies. This Statewide stroke disease database will create an exchange meant for sharing examples, ideas and techniques for informing policy and documenting geographical disparity, as it is now demonstrated in the western world such as the United States to reduce the prevalence of stroke. Also, population registry and property registry are linked together in Sweden so as to provide data for calculating distance network [7, 8]. Geographical Information System (GIS) are increasingly used to analyze geographical distribution of disease as well as relationships with causative agents and their geographical environment [9]. Extensive review of previous studies has shown that even disease like stroke may have geographical influence and more accurate analysis using GIS technique can be generated. In the review of studies of geographies of declining stroke morbidity in the United States [10], it shows that such variation is typical of other countries.

**Figure 1:**
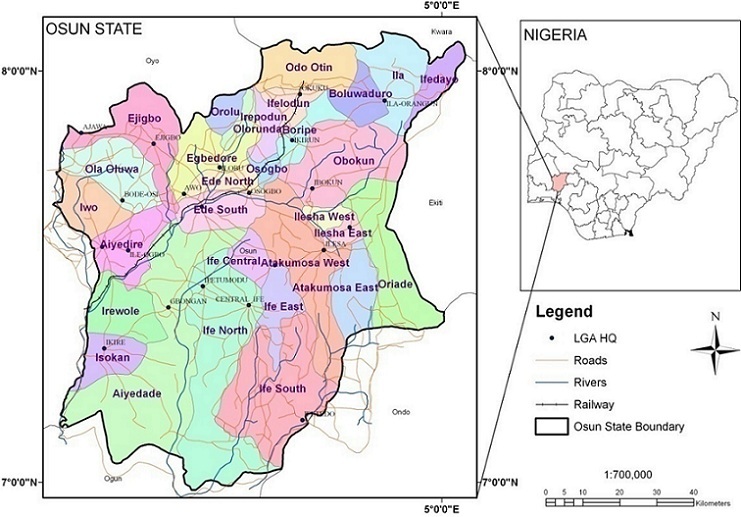
The study area showing its location within Nigeria

The result show that the trend varies between age and sex groups in the population and that particularly with older people. Overall health gain across the country is being achieved at the cost of greater inequality in health between areas. Osun is one of the States that have in recent time experience high rate of stroke disease. There are no information on geographical disparities for stroke, the trends of stroke incidence and prevalence in the study area. This study attempts to uncover the spatial pattern of diagnosed stroke disease reflecting socio-demographic characteristics in Osun State using GIS technique. Osun State is regarded as the origin of Yoruba culture, industry and of people of Yoruba. The State is divided into 30 local government areas (Figure 1). Administrative set-up of the state is divided into three senatorial districts: Osun I (West), Osun II (Central) and Osun III (East). Each of these districts is further divided into two zones, making a total of six zones. Ede and Iwo zones make up Osun I, whereas Osun II comprises of Osogbo and Ikirun zones. Ilesa and Ife zones make up Osun III senatorial districts. The Population of the State is about 3,416,959 [11]. The urban centres namely, Osogbo, llesha, lle-ife, ljebuJesa, Ejigbo, Modakeke, lfetedo, Ede, lkirun, Ipetu-ljesa, lla and Ode-Omu all had relatively large populations and these urban areas are located within the upper half of the state. The smaller towns and villages constitute (Rural) the lower half. With the creation of Osun State in 1991, there has been an influx of people into the state, especially civil servants moving into Osogbo the state capital and other neighbouring settlement such as, Ede, Gbongan, Ikirun. Osun State being a part of the cocoa belt is a major destination for migrant farmers from other parts of Nigeria. Some of the migrants work as hired labourers in cocoa farms whereas others settle down as migrant's tenant farmers and traders. The major sub-ethnic groups in Osun State are Ife, Ijesha, Oyo, Ibolo and Igbomina of the Yoruba people, although there are also people from other parts of Nigeria. The study aims to examine the spatial pattern of stroke incidence and the associated factors in Osun State. This study could help health care planners and other organizations to focus on the main problems of a community rather than of individual patients and to identify measures for improving the cardiovascular health of the community as a whole.

## Methods

The administrative map of Osun State was obtained from ministry of Lands and Physical Planning, Survey Department, Osogbo. Field work was conducted to capture Global Positioning System (GPS) coordinates of 10 major participating hospitals capable of delivering acute stroke care in the study area. Ministry of Health Osogbo was contacted for the list of Major Hospitals in the State. Attribute information about stroke patients between 1990 and 2012 were extracted from inpatients records obtained from the participating hospitals. Demographic and population data for the 30 Osun State Local Government Areas were obtained from the openly available 2006 census from the National Population Commission (NPC). Inpatients records from Obafemi Awolowo University Teaching Hospital (OAUTHC), Ile Ife the main referral hospital in Osun State and nine other main hospitals in the State, was generalized to the rest of the population in Osun State giving a total of 1,750 patients records. Medical records were reviewed for the following attributes as variables: Diagnoses, address, year of admission, history of previous stroke, alcohol or tobacco use, Age, Sex, family history, underlying disease, Occupation, Religion, and ethnicity. About 87.77% of the 1,750 medical records are with complete street address, 6.05%, fall into other states, 4.17% had missing or inadequate address, and about 2% fall under 30 years age range and were therefore, excluded in the analysis. This was because stroke is not common within that age bracket. Majority of the patients records examined did not contain relevant information such as family history, history of previous stroke, alcohol or tobacco use and level of patient income.

There was also no adequate data for socio-economic profiles for sampled stroke patients, such as; household income, education level, family history, population below poverty level and environmental. Also, physiological characteristics such as, physical inactivity of patients sampled were unknown. Hospital based data collected retrospectively were linked to population catchment areas served by the 10 health care facilities involved in the management of stroke patients in the State. Also, the 2006 population data acquired was at this stage projected for 2007-2012 using the formula [11]: Pt = Po e r t (eq.1); Pt = Population to be projected (current Population); Po = Population for the base year (Year from which we are projecting from); e = Exponential functions; r = Growth Rate in Percentage (given as 3.2% for the current growth rate); t = Period in year(s). A statewide stroke incidence information database was developed using MS-Excel 2007 software. This was used to examine stroke patient admission from 1990 to 2012. Patient's addresses were grouped according to local government areas, in order not to expose patient's rooftop address in conformance with medical ethics rules. The existing analog administrative map of Osun State was scanned into GIS, geo-referenced, digitized and shapefiles were created for; Osun boundary, local government areas, settlements, and other features. GPS coordinates of participating health care facilities were overlaid on the state administrative map to show spatial distribution in GIS environment. The geographical coordinates of GPS Point symbols were used to represent the hospitals. Calculations were made to determine stroke incidence rate and prevalence in the area. The 2006 population figure was used to project the population of people that falls within 30 years age bracket and above for 2007 to 2012 in Osun State. The total population (Po) of that age group in 2006 was 1, 109,912 which was about 33% of the total population for that year and 3.2% growth rate [11] were used to get the projected population for the years required. The detailed projected figures and computed incidence rates are shown in Table 1. Incidence rate for stroke in the study area was calculated using: number of new stroke cases for required year/Population at risk × 100%.

**Table 1:** Projected population and incidence rates per 100,000 population in Osun

Year	No of patients	Age adjusted incidence rate per 100,000 population	Projected population (age 30& above ) using 2006 census
Jan-Dec 1990	32	2.88	------
Jan-Dec 1991	33	2.97	-----
Jan-Dec 1992	33	2.97	-------
Jan-Dec 1993	37	3.33	------
Jan-Dec 1994	38	3.42	------
Jan-Dec 1995	41	3.69	------
Jan-Dec 1996	45	4.05	------
Jan-Dec 1997	46	4.14	------
Jan-Dec 1998	46	4.14	------
Jan-Dec 1999	49	4.41	------
Jan-Dec 2000	56	5.04	-----
Jan-Dec 2001	60	5.4	------
Jan-Dec 2002	62	5.58	-----
Jan-Dec 2003	66	5.94	-----
Jan-Dec 2004	70	6.3	------
Jan-Dec 2005	75	6.75	-----
Jan-Dec 2006	81	7.29	-----
Jan-Dec 2007	100	8.72	1,146,003
Jan-Dec 2008	108	9.12	1,183,268
Jan-Dec 2009	108	8.83	1,221,745
Jan-Dec 2010	113	8.95	1,261,473
Jan-Dec 2011	118	9.05	1,302,493
Jan-Dec 2012	119	8.84	1,344,847
Total	1,536		

## Results

**Figure 2:**
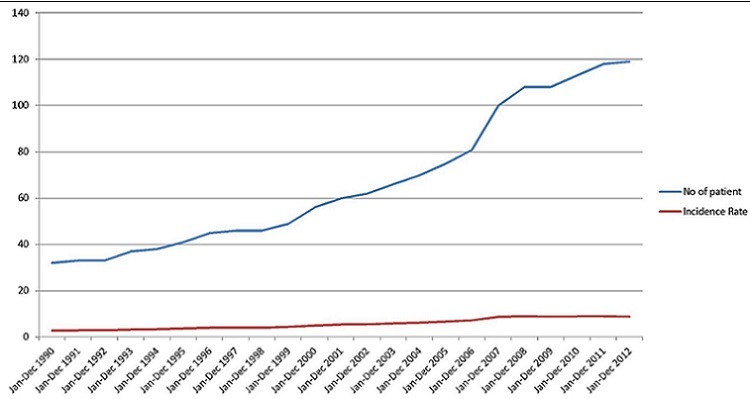
Incidence rate per 100,000 populations among age groups 30years and above

**Figure 3:**
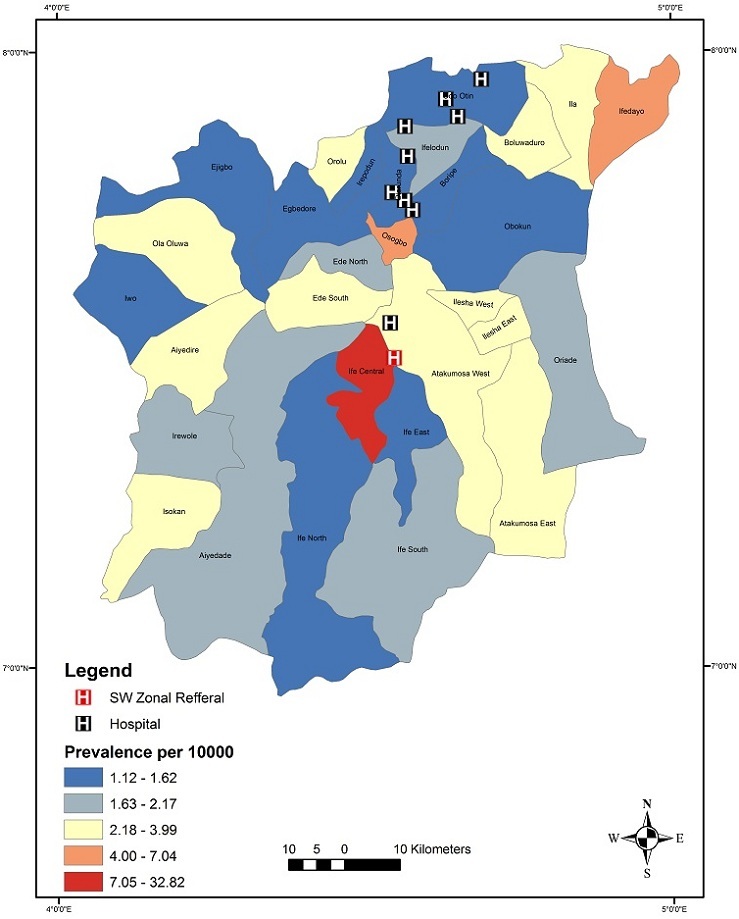
A spatial distribution of stroke prevalence Rate per 10,000 populations within the LGAs in Osun State

**Figure 4:**
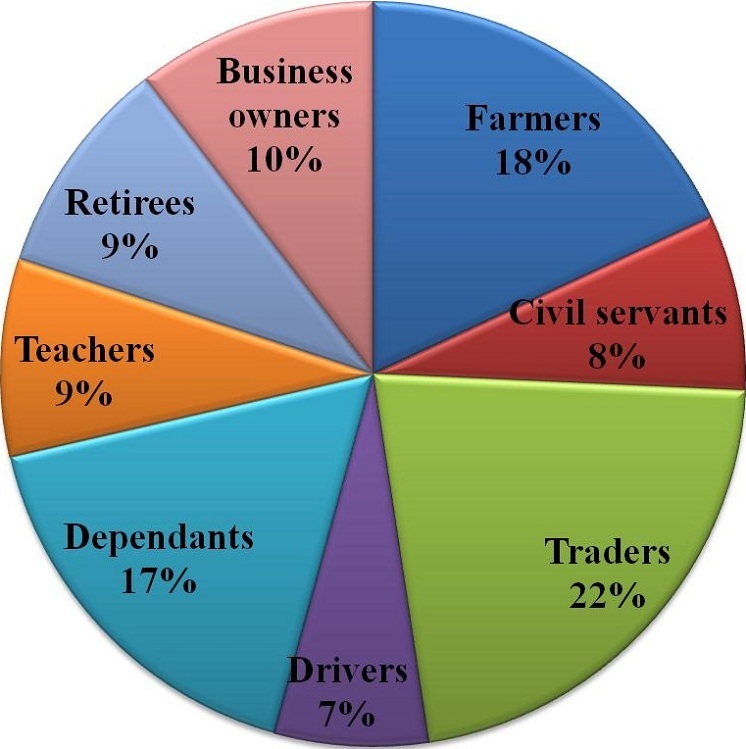
Variation of stroke disease per occupation among sampled patients

**Figure 5:**
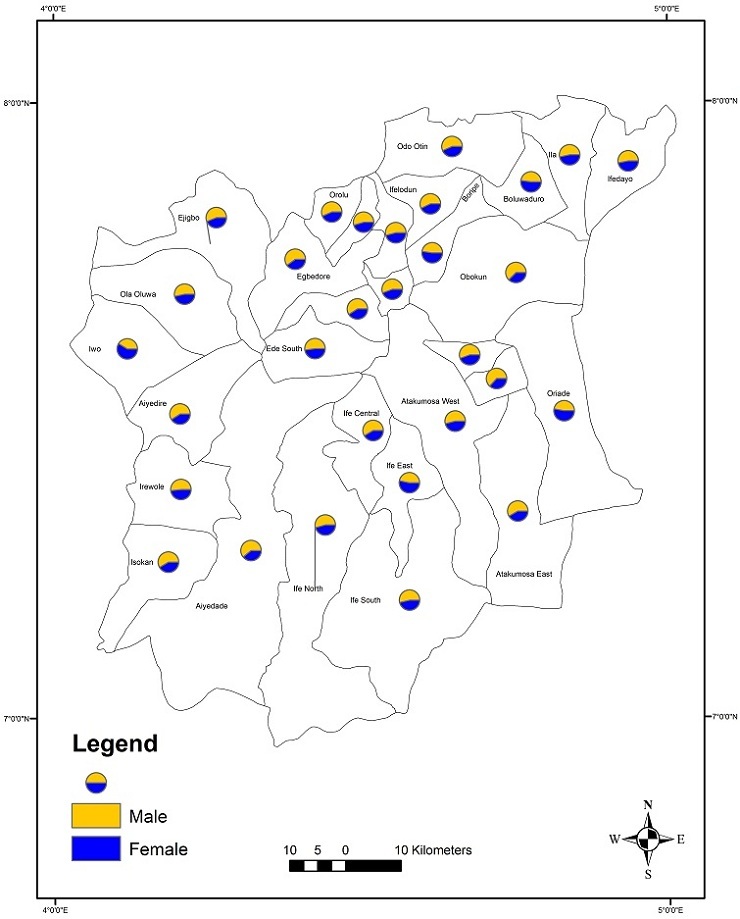
Male to female ratio for stroke patients

**Figure 6:**
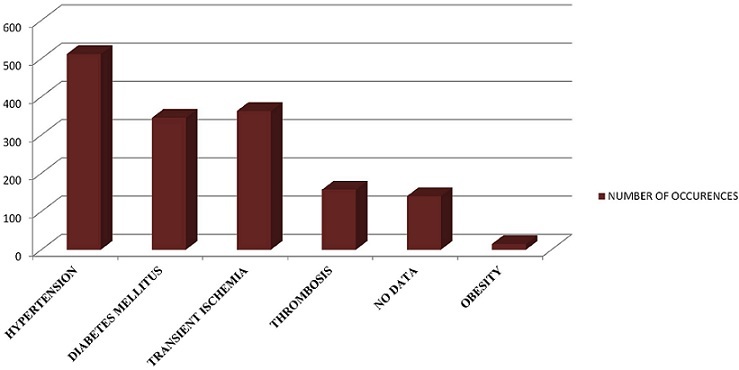
Underlying diseases among sample patient

Results indicated a slight increase in the annual incident rates of stroke in Osun State. Annual incidence rates increased from 3 per 100,000 persons-year in 1990 to 9 per 100,000 persons-year in 2012 (Figure 2). Prevalence was used as a measure to evaluate the burden of stroke disease in Osun State. It is given by existing cases between 1990 and 2012 divided by the defined population at that time. This was computed for each local government area in the State. Table 2 shows the result obtained. This presents a snapshot of Stroke disease in the local government areas in Osun State (Figure 3). Osun East senatorial district was seen to have the highest prevalence rate (Figure 3) with only two hospitals followed by Osun Central with 8 main hospitals. Osun West has no main Hospital and therefore had the lowest rate. Analysis at local government levels identified Ife central as the area with the highest prevalence rate (Figure 3) followed by Ifedayo and Osogbo LGAs respectively. Spatial analysis of the disease was carried out with respect to gender, occupation, and age range. The disease is rampant with traders (18%) while drivers are lowest with 7% (Figure 4). Figure 5 show that males aged between 59 and 69 had the highest incident of stroke. Males were seen to be higher than Females when considered. With the total numbers of patients sampled, male was about 56.4% to 43.6% female. However, when we estimated using total female and male population crude (all ages) male is 49.8% to 50.2% Female. With adjusted population (30 years and above), males were slightly higher, that is 50% males to 49% females. This study reveals that “30-69 years age range constituted 70.8% of the patient's population”. This supports previous findings that the prevalence of stroke is higher among the working class population in Nigeria [2]. Analysis of underlying disease among sampled patients (Figure 6) revealed a trend of high levels of hypertension, and diabetes mellitus in the highest risk LGAs of Osun. The major predisposing risk factors are therefore, hypertension and diabetes mellitus.

**Table 2:** Thirty (30) years and above Prevalence Rates per 10000 population in each LGAs in Osun

Osun LGAs	2012 Projected population	2006 population census	30yrs and above total for Both Sex	Total Nos. of patients (Both Sex)	Nos. OfFemale Patients	Nos. Of Male Patients	Prevalence rate /100,000 population for 30yrs and above (1990-2012)
Aiyedade	181, 228.3476	149,569	49,873	36	14	22	72.18
Aiyedire	92, 461.3654	76,309	24,878	22	9	13	88.4
Atakumosa East	92,214.1847	76,105	25,750	24	10	14	93.2
Atakumosa West	82, 817.6798	68,350	23,248	24	11	13	103.2
Boluwaduro	85,972.8698	70,954	22,892	23	12	11	100.4
Boripe	168,109.5909	138,742	43,915	21	11	10	47.8
Ede North	101,559.7994	83,818	26,464	22	9	13	83.1
Ede South	91,467.7956	75,489	23,968	23	11	12	96.0
Egbedore	89,626.0564	73,969	24,042	10	4	6	41.6
Ejigbo	160,564.5186	135,515	43,155	25	11	14	57.9
Ife Central	202,596.1571	167,204	50,942	665	275	390	1305.4
Ife East	228,538.0229	188,614	60,630	30	16	14	49.4
Ife North	185,717.5868	153,274	50,525	24	11	13	47.5
Ife South	162,957.5709	134,490	46,250	28	13	15	60.5
Ifedayo	45,447.3373	37,508	12,457	32	15	17	256.8
Ifelodun	116,858.3513	96,444	30,952	21	9	12	67.8
Ila	75,189.0023	62,054	20,291	30	14	16	147.8
Ilesha East	127,729.4592	105,416	35,037	38	14	24	108.4
Ilesha West	129,417.3163	106,809	36,178	36	16	20	99.5
Irepodun	144,903.6771	119,590	37,039	22	10	12	59.3
Irewole	173,033.8199	142,806	47,112	29	14	15	61.5
Isokan	123,663.0937	102,060	33,401	29	12	17	86.8
Iwo	231,850.7301	191,348	62,222	37	17	20	59.4
Obokun	141,583.6999	116,850	37,589	23	9	14	61.1
Odo Otin	169,035.0815	132,078	41,429	22	10	12	53.1
Ola Oluwa	92,362.0085	76,227	25,234	28	13	15	110.9
Olorunda	159,515.2119	131,649	42,373	24	11	13	56.6
Oriade	179,788.4596	148,379	48,648	34	18	16	69.8
Orolu	124,598.5024	102,832	33,271	30	13	17	90.1
Osogbo	188,423.2471	155,507	50,143	124	55	69	247.3
Total	4,149,615.00	3,416,959	1,109,912	1,536	669	867	

## Discussion

The focus of this paper is on the identified high risk areas for cardiovascular disease and healthcare facilities available for the management of the disease within Osun State. The study covers a spatial analysis of the distribution of diagnosed stroke cases in relation to their underlying population. The study was based on diagnosed cases from 1990 to 2012 acquired from ten available hospitals participating in delivering acute stroke care in the State. Patients that were handled locally or with herbal approach and those who could not make it to the ten recognized hospitals in the case study area within the study period are not captured by this effort. It can be presumed that many stroke patients with symptoms of stroke were admitted to other hospitals especially when these hospitals were closer to them than the ten from which the data for this study was drawn. However, it is expected that these patients would be referred to any of the 10 hospitals for further treatment and as such their data would be captured in this study. But we can't rule out the possibility that some stroke patients never made it to any hospital. Therefore, the task of evaluating the spatial pattern of stroke disease within Osun State has been limited to diagnosed stroke cases as the other undiagnosed cases are difficult to attain without a population-based registry for stroke and other heart diseases. This study highlights the importance in the identification and evaluation of high risk areas of stroke using GIS techniques and thus provides insights to public health measures that can reduce the burden in Osun. This study advances our knowledge in this area by identifying the geographical disparities in the incidence of diagnosed Stroke disease within the study area. The outcome of the study identified an annual increase in crude stroke incident rates after estimating for the year 2012 with a growth rate of 3.2%. According to our findings, the number of new cases for stroke in Osun hospitals is continually increasing since the 1990s above estimated growth rate of 3.2%. If population wide intervention is not applied to the increasing incident rate of stroke, 40% of the burden is projected to rise from around 38 million Disability-Adjusted Life Years (DALYs) globally in 1990 - 61 million DALYs in 2020, therefore, urgent attention is needed [12]. Given that stroke is uncommon among age group less than 30 years. This study indicated that stroke was more prevalent among those aged 75 years and older in Osun State. Furthermore, the trend was found to be high in adults of productive ages that contribute to the work force in Osun State, Nigeria (30 - 69 years).

Studies indicate that sex discrepancies occur in patients with stroke as more males than females are diagnosed with stroke [13]. The result from this study also supports previous finding as males were approximately 56.4% and females 43.6%. However, when we estimated using total female and male population crude (all ages), males were 49.8% and females 50.2%. Furthermore, this study showed that, females were slightly younger than males therefore, using an adjusted population (30 years and above), males were slightly higher at 50% while females were 49%. The study also indicated that there were clustering of determinants of stroke at Ife Central, followed by Osogbo and Ifedayo Local Government Areas. The study indicated that the increase is typical of urban settlements, as these areas tended to have high proportions of the population. The burden varied by geographical location while Hypertension and Diabetes Mellitus were common among patients in the highest risk areas. Incidence of stroke was found to be widespread among traders, farmers and dependents (unemployed). Moreover, disparities exist in the distribution of health care facilities involved in the management of stroke patients, they are found mostly in the urban areas. The stroke patterns raise some questions on the spatial distribution of the disease within the state. The foremost question has to do with the low number of cases registered within the rural areas as opposed to the urban areas. It can be argued that access to vital information needed in identifying stroke patients as well as available options for stroke healthcare are not being accessed by rural dwellers that might have the disease. However, the proportion of stroke distribution between the urban and rural areas can only be addressed with extensive investigations in order to draw out reasonable conclusions on the factors behind this pattern.

## Conclusion

Statewide mapping of stroke incidence has reduced prevalence in developed countries due to life style modification and adequate health care planning [12]. Stroke disease in Osun State has been investigated in this study using a spatial approach. A combination of maps, charts and table has been used to establish that: (i) the prevalence of stroke is higher among those above 70 years of age in Osun State. (ii) Social inequalities in stroke are persistence as incidence rates in urban areas (North) are around 3 times higher than in the rural area (South). (iii) There is a variation in the distribution of health care facilities involved in administering care to stroke patients in Osun State. Primary prevention can be realized statewide only by modest reduction of early life blood pressure of the entire population. Role of rural clinics is also important, stroke awareness and consequent improved recognition, removal of associated stigma must be obtained through the same means for instance, the FAST- Face, Arm, Speech Test. Moreover training healthcare workers on the recognition and essential management of stroke also needs to be developed and implemented on a progressively large scale. This study is an important start for prospective stroke disease surveillance across Osun area, useful for identifying high risk Local Government areas and improving population health programs aimed at addressing health disparities and improving population health. To our knowledge, this is the first study to investigate spatial pattern and clusters of stroke risk to better understand observed disparities and identify specific health needs at the local government level to aid population health planning. Only through monitoring that is possible in a Statewide Stroke Incidence Information Registries can accurate data on stroke burden be acquired, which offers entry point for local government to register all stroke patients in Health Information System (HIS) and Land Information System (LIS). With more data becoming available, adjustments may be applied. Studies have shown that death rates from cardiovascular disease (CVD) have decreased in developed countries. For example, The American heart association reported that the relative rate of death attributable to CVD declined by 32.7% from 1999 to 2009 [14]. Therefore, there is a large potential to reduce the population burden of this disease in Osun State. Public health measures that can reduce the burden of stroke can only be achieved through the monitoring of a state-wide stroke incidence information database. The end product of such state-wide stroke incidence information database would be an accurate data on stroke burden. Better knowledge of stroke etiology will inform policy, encourage primary preventions and other intervention measures.

### What is known about this topic

- There is an increasing incident rate of stroke disease in Nigeria;
- Stoke is the third highest cause of death in Nigeria;
- There is evidence of geographical disparities for stroke disease worldwide but little is known within Nigeria.

### What this study adds

- The study improves on the knowledge of the spatial distribution of diagnosed stroke cases within Nigeria;
- Spatial inequalities in the access to Stroke healthcare is a concern that needs to be addressed in order to manage the disease adequately.
